# Association between cellular characteristics and clinical outcomes following autologous micro-fragmented adipose tissue injection for knee osteoarthritis

**DOI:** 10.3389/fbioe.2026.1843722

**Published:** 2026-06-01

**Authors:** Chongyi Fan, Zhenda Zhao, Boran Liang, Yugang Xing, Shuming Li, Kai Xiao, Jing Chen

**Affiliations:** Department of Orthopaedic Surgery, Aerospace Center Hospital, Beijing, China

**Keywords:** autologous micro-fragmented adipose tissue (aMFAT), cell viability, knee osteoarthritis, regenerative medicine, viable nucleated cell load

## Abstract

**Background:**

Autologous micro-fragmented adipose tissue (aMFAT) has emerged as a promising orthobiologic therapy for knee osteoarthritis (KOA). However, the relationship between the cellular characteristics of aMFAT products and clinical outcomes remains unclear.

**Methods:**

This retrospective single-center study included 40 patients with symptomatic KOA (Kellgren–Lawrence grade II–III) who received a single intra-articular injection of aMFAT. Clinical outcomes were assessed using the Western Ontario and McMaster Universities Osteoarthritis Index (WOMAC) at baseline, 3 months, and 6 months. Cytological analyses were performed in a subgroup of 14 patients, including cell viability, total nucleated cell count, viable nucleated cell load, and cell subpopulation composition. Subgroup and correlation analyses were conducted to explore associations between cellular characteristics and clinical outcomes.

**Results:**

Significant improvements in WOMAC total and subscale scores were observed at both 3 and 6 months compared with baseline (P < 0.05). At 6 months, 97.5% of patients achieved the minimal clinically important difference. In the cytological subgroup, both cell viability and viable nucleated cell load were positively correlated with clinical improvement, with viable nucleated cell load demonstrating a stronger association (r = 0.93 vs. 0.84). Patients with higher cell load showed significantly greater and more sustained improvements. Fibroblast proportion was positively correlated with clinical outcomes, while no significant association was found for MSCs or macrophages. No serious adverse events were reported.

**Conclusion:**

A single intra-articular injection of aMFAT was associated with short-term clinical improvement and a favorable safety profile in patients with knee osteoarthritis. Clinical outcomes showed exploratory associations with cellular characteristics, particularly viable nucleated cell load. These findings require validation in future studies.

## 1 Introduction

Knee osteoarthritis (KOA) is one of the most common degenerative joint diseases, characterized by articular cartilage degeneration, synovial inflammation, and subchondral bone structural changes (Kloppenburg et al., 2025). With the aging population and increasing prevalence of obesity, the incidence of KOA continues to rise, posing a substantial burden on global public health systems (Yang et al., 2023).

Current treatment strategies for KOA generally follow a stepwise management approach. Early-stage patients are mainly treated with non-surgical interventions, whereas patients with end-stage disease can achieve reliable pain relief and functional improvement through total knee arthroplasty (TKA) (Arden et al., 2021). However, for patients in the intermediate stage (Kellgren–Lawrence grade II–III), conventional conservative treatments are often insufficient to effectively delay disease progression, while joint replacement is not yet an appropriate option. Therefore, there remains a lack of ideal treatment strategies for this population (Arden et al., 2021; Bannuru et al., 2019).

In recent years, advances in regenerative medicine have provided new therapeutic approaches for osteoarthritis, among which cell-based therapies represented by mesenchymal stem cells (MSCs) have attracted considerable attention. MSCs can secrete a variety of anti-inflammatory cytokines and growth factors through paracrine mechanisms, thereby modulating intra-articular inflammation and promoting cartilage repair (Zha et al., 2021; Kim et al., 2023; Sadeghirad et al., 2024; Jin et al., 2025). Several clinical studies have reported that MSC therapy can improve pain and joint function in patients with KOA (Kim et al., 2023; Sadeghirad et al., 2024; Jin et al., 2025). However, MSC therapy typically relies on *in vitro* expansion, which involves complex procedures, high costs, and strict regulatory requirements, thereby limiting its clinical application.

Adipose-derived orthobiologic therapies have emerged as a promising alternative to overcome these limitations. Among them, stromal vascular fraction (SVF) represents a heterogeneous cell population obtained from adipose tissue through enzymatic or mechanical processing, containing adipose-derived stromal cells, endothelial cells, pericytes, and other cellular components. SVF has been reported to exert anti-inflammatory and tissue repair effects through the secretion of various bioactive factors (De Francesco et al., 2021; Bora and Majumdar, 2017; Boada-Pladellorens et al., 2022; Goncharov et al., 2023). Previous studies have reported the potential efficacy of SVF in the treatment of knee osteoarthritis. For example, Vargel et al. demonstrated that intra-articular injection of SVF significantly improved pain and functional scores in patients with KOA (Malanga et al., 2021). In addition, systematic reviews have indicated that adipose-derived orthobiologic products have certain clinical value in improving KOA symptoms and exhibit favorable safety profiles (Malanga et al., 2021; Hudetz et al., 2017; Varg et al., 2022; Onorato et al., 2024; Richter et al., 2025; Hohmann et al., 2025; Ulivi et al., 2023).

Although autologous micro-fragmented adipose tissue (aMFAT) shares certain biological features with SVF (Wang et al., 2025), it is a mechanically processed product that preserves the native extracellular matrix architecture, distinguishing it from enzymatically derived SVF (de Girolamo et al., 2023). In clinical practice, aMFAT products may exhibit substantial heterogeneity in cellular composition, cell viability, and cell number due to individual patient differences and processing techniques (Bora and Majumdar, 2017; Boada-Pladellorens et al., 2022; Goncharov et al., 2023). However, there is limited evidence regarding whether these cellular characteristics influence clinical outcomes.

Therefore, this study retrospectively analyzed the clinical data of patients with symptomatic knee osteoarthritis who received intra-articular injection of aMFAT. While evaluating clinical efficacy and safety, cytological analyses were performed on adipose micro-fragmented products from a subset of patients to further investigate the association between cell viability, viable nucleated cell load, and clinical outcomes.

Given the heterogeneity of orthobiologic therapies and the lack of high-level evidence, this study was designed as an exploratory analysis rather than to establish causal or predictive relationships.

## 2 Materials and methods

### 2.1 Study population

This was a retrospective, observational, single-center study without a control group. A total of 40 consecutive knee cases from patients with symptomatic knee osteoarthritis who received a single intra-articular injection of autologous micro-fragmented adipose tissue (aMFAT) (Lipogems® Ortho Kit, Lipogems International SpA, Milan, Italy) between October 2024 and October 2025 were included. All patients provided written informed consent. The study was approved by the Ethics Committee of Aerospace Center Hospital (approval date: 11 November 2025; approval number: 2025-115). This study was conducted as a retrospective chart review. All patients received treatment as part of routine clinical practice prior to ethics approval. Ethical approval was obtained before data collection and analysis were performed.

Inclusion criteria: (1) Age 50–75 years; (2) Symptomatic knee osteoarthritis confirmed by X-ray or MRI (Kellgren–Lawrence grade II–III) with symptoms lasting ≥3 months; (3) Poor response to conservative treatment within the previous 3 months (e.g., NSAIDs, physical therapy, hyaluronic acid, or corticosteroid injections).

Exclusion criteria: (1) Intra-articular injection within the past 3 months; (2) Rheumatoid arthritis; (3) Active or prior knee joint infection; (4) History of open knee fracture; (5) Recent trauma; (6) Severe coronal deformity (hip–knee–ankle angle >10° on weight-bearing radiographs); (7) History of malignancy (e.g., tumors or hematological diseases); (8) Systemic immune diseases; (9) BMI <19 or insufficient abdominal adipose tissue for harvesting.

### 2.2 Surgical procedure

Adipose tissue was harvested from the abdomen in all patients, and the procedure was performed by plastic surgeons or orthopedic residents.

**FIGURE 1:**
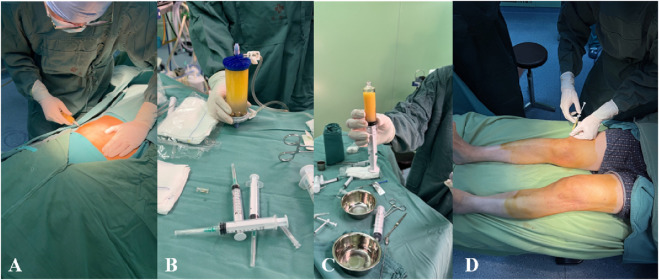
**(A)** Under strict aseptic conditions, the abdominal donor site was selected. After local anesthesia, tumescent anesthesia was administered. Adipose tissue was then harvested using a blunt-tip cannula connected to a negative-pressure aspiration device, applying a low-pressure, low-shear technique to gently aspirate the fat. **(B)** The harvested adipose tissue was placed into a sterile container and subjected to sedimentation or gentle washing to remove free oil, blood, and residual anesthetic solution. **(C)** The pretreated adipose tissue was transferred into the Lipogems processing device, where it was processed within a closed sterile system. **(D)** The prepared micro-fragmented adipose tissue suspension was loaded into a sterile syringe and injected into the target joint cavity.

The aMFAT procedure was performed under local anesthesia using the Lipogems® device (Italy) (Baria et al., 2024). After harvesting, adipose tissue was processed according to the manufacturer’s protocol (as previously described in the literature). A standardized procedure was applied to all patients to ensure: (1) Use of the same device; (2) Consistent processing steps. Approximately 8 mL of aMFAT was injected into each knee joint (Figure 1).

Some patients received unilateral injections, while others received bilateral injections. In cases of bilateral osteoarthritis, only the more symptomatic knee was analyzed to avoid statistical bias caused by repeated measurements from the same patient. Therefore, the unit of analysis in this study was the patient rather than the knee joint, with each patient contributing only one data point.

### 2.3 Postoperative rehabilitation

After injection, passive knee flexion–extension exercises were performed 10 times. Patients were allowed to ambulate starting on postoperative day 1. Daily activities were gradually resumed within 1 week as tolerated. High-impact activities were avoided during the first 6 weeks postoperatively.

### 2.4 Cytological assessment

All laboratory analyses were performed by Beijing Ruijian Biomedical Company.

**FIGURE 2:**
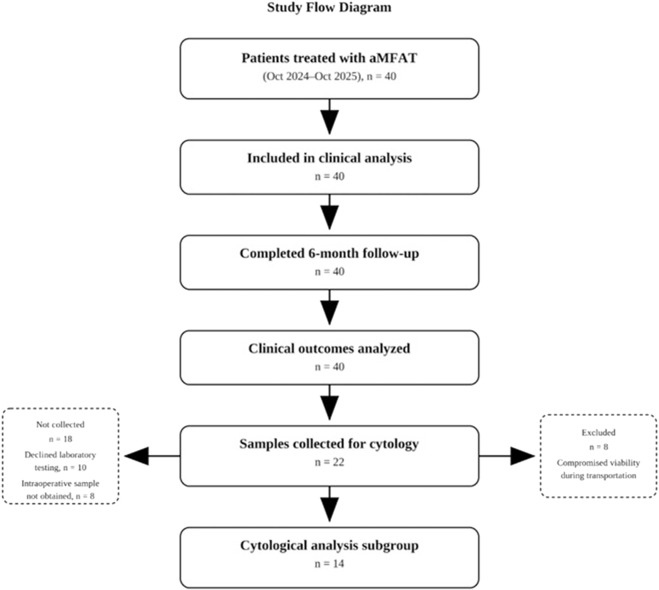
Flowchart illustrating patient inclusion, follow-up, and the cytological analysis process. A total of 40 patients received aMFAT treatment and were included in the clinical analysis, all of whom completed the 6-month follow-up. Cytological samples were collected from 22 patients. Among the remaining patients, 10 declined laboratory testing and 8 did not undergo intraoperative sample collection. Of the collected samples, 8 were excluded due to compromised viability during transportation, resulting in a final cytological analysis subgroup of 14 patients. aMFAT, autologous micro-fragmented adipose tissue.

Cytological analyses were performed in a subset of 14 patients based on sample availability and logistical feasibility during the study period (Figure 2). These patients were not randomly selected, which may introduce potential selection bias.

#### 2.4.1 Total nucleated cell count and viability

Adipose micro-fragment samples were enzymatically dissociated, and total nucleated cell counts were measured using an automated cell counter. Results were expressed as the number of nucleated cells per gram of adipose tissue (cells/g).

Adipose tissue was digested with enzyme solution to obtain a single-cell suspension. The suspension was filtered through a 100 μm cell strainer, followed by centrifugation at 300 *g* for 10 min. After removal of the supernatant, red blood cells were lysed. The sample was then filtered through a 30 μm cell strainer and centrifuged again. The final cell pellet was resuspended in flow cytometry staining buffer, and cell counting with viability assessment was performed using imaging-based analysis.

Cell viability was defined as the percentage of viable cells among total nucleated cells.

#### 2.4.2 Calculation of viable nucleated cell load

The viable nucleated cell load was calculated as follows:

Viable nucleated cells per gram (cells/g) = total nucleated cells per gram (cells/g) × cell viability (%).

#### 2.4.3 Flow cytometric analysis of cell subpopulations

In patients who underwent cytological testing, cell subpopulations in aMFAT-derived samples were analyzed by flow cytometry. Following enzymatic digestion to obtain a single-cell suspension, cells were stained with fluorescently labeled antibodies and analyzed using a flow cytometer. Cell viability was assessed using Calcein AM staining.

Major cell populations were identified using predefined marker combinations, including mesenchymal stromal cells (CD45−CD31−CD105+), T lymphocytes (CD45+CD3+), macrophages (CD45+CD3−CD68+), and fibroblasts (CD45−CD140b+). Gating strategies were established based on forward and side scatter characteristics, viability staining, and marker expression, with unstained samples used as controls to define positivity thresholds.

The proportion of each subpopulation was expressed as a percentage of total nucleated cells and used for subsequent correlation analyses. Standardized staining and acquisition procedures were applied to ensure consistency across samples.

### 2.5 Subgroup analysis based on cell viability and viable nucleated cell load

A total of 14 patients underwent cytological testing. Patients were divided into high and low groups based on the median values of cell viability and viable nucleated cell load. Subgroup analyses were performed to evaluate the association between these cellular parameters and clinical outcomes. The use of median-based subgrouping was intended for descriptive comparison; however, given the small sample size, analyses based on continuous variables were considered more informative.

### 2.6 Clinical outcome assessment

#### 2.6.1 Baseline data collection

The following baseline characteristics were collected: (1) Age; (2) Sex; (3) Body mass index (BMI); (4) Kellgren–Lawrence (KL) grade.

#### 2.6.2 Clinical outcomes

All patients were evaluated using the Western Ontario and McMaster Universities Osteoarthritis Index (WOMAC), including pain, stiffness, and function subscales, at baseline and during follow-up.

Follow-up assessments were conducted at 3 and 6 months postoperatively, with the day of treatment defined as baseline. Follow-up data were collected through outpatient visits, electronic questionnaires, and telephone interviews. Adverse events were recorded, including type, severity, and duration. WOMAC scores were assessed using a standardized questionnaire at all time points.

Primary outcome: (1) Change in total WOMAC score from baseline to 6 months. (2) Based on previous studies, an improvement of ≥12 points was defined as the minimal clinically important difference (MCID).

Secondary outcomes: (1) Changes in WOMAC subscale scores at 3 and 6 months; (2) Incidence of adverse events; (3) Subgroup analysis based on cell viability and viable nucleated cell load.

### 2.7 Statistical analysis

Statistical analysis was performed using SPSS software (version 26.0, IBM Corp., Armonk, NY, United States). Normality of continuous variables was assessed using the Shapiro–Wilk test. Normally distributed data were expressed as mean ± standard deviation (SD), while non-normally distributed data were presented as median (interquartile range). Categorical variables were expressed as frequencies and percentages. Repeated measures ANOVA or the Friedman test was used to compare WOMAC scores at baseline, 3 months, and 6 months. When overall differences were statistically significant, pairwise comparisons were performed with Bonferroni correction. Multivariable regression analyses were performed to adjust for potential confounders, including age, sex, BMI, Kellgren–Lawrence grade, and baseline WOMAC scores.

Patients were divided into groups based on the median values of cell viability and viable nucleated cell load. Between-group comparisons were conducted using the independent samples t-test or Mann–Whitney U test. Spearman rank correlation analysis was used to evaluate the association between cellular parameters and clinical outcomes. All statistical tests were two-tailed, and a P value <0.05 was considered statistically significant.

## 3 Results

### 3.1 Baseline characteristics

A total of 40 patients with symptomatic knee osteoarthritis who met the inclusion criteria were included in this study. Baseline characteristics are summarized in Table 1. The mean age of the patients was 60.2 ± 4.4 years, including 29 females (72.5%) and 11 males (27.5%), with a mean body mass index (BMI) of 26.4 ± 2.5 kg/m^2^. According to the Kellgren–Lawrence (KL) classification, 17 patients (42.5%) were classified as grade II and 23 patients (57.5%) as grade III. All patients completed the 3- and 6-month follow-ups. The baseline WOMAC total score was 58.6 ± 4.7.

**TABLE 1:** Baseline characteristics of the study population (n = 40).

Variable	Value
Age (years)	60.2 ± 4.4
Sex, n (%)	
Male	11 (27.5%)
Female	29 (72.5%)
BMI (kg/m^2^)	26.4 ± 2.5
KL grade, n (%)	
KL grade II	17 (42.5%)
KL grade III	23 (57.5%)
Baseline score	
WOMAC total	58.6 ± 4.7
Pain	12.2 ± 1.2
Stiffness	4.7 ± 0.6
Function	42.0 ± 3.2

Data are presented as mean ± SD, or n (%). Abbreviations: BMI, body mass index; KL, Kellgren–Lawrence; WOMAC, Western Ontario and McMaster Universities Osteoarthritis Index.

### 3.2 Clinical outcomes

#### 3.2.1 Primary outcome

**FIGURE 3:**
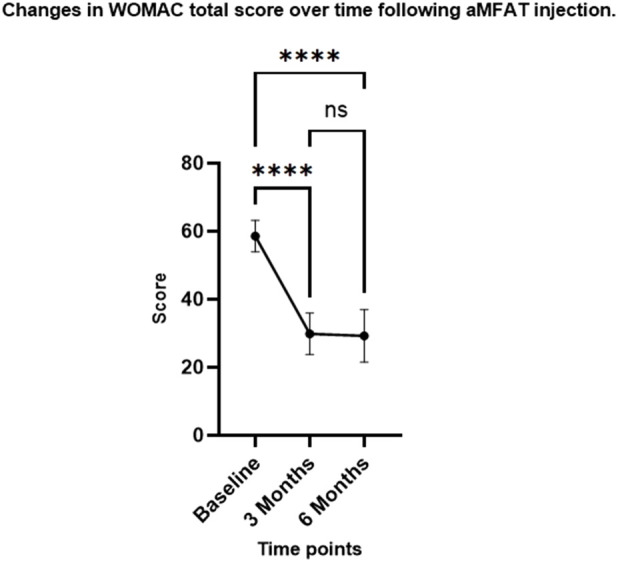
WOMAC total score improved significantly at 3 and 6 months compared with baseline (P < 0.001), with no significant difference between 3 and 6 months.

Compared with baseline, WOMAC total scores were significantly reduced at both 3 and 6 months post-treatment (Table 2). The mean WOMAC total score decreased from 58.6 ± 4.7 at baseline to 29.9 ± 6.1 at 3 months and 29.2 ± 7.7 at 6 months. The mean improvement from baseline to 6 months was 29.4 ± 8.0. There was no statistically significant difference between 3 and 6 months (P > 0.05) (Figure 3).

**TABLE 2:** Changes in WOMAC Scores and Clinical Improvement Outcomes Following aMFAT Treatment.

Outcome	Baseline	3 Months	6 Months	P value
WOMAC total	58.6 ± 4.7	29.9 ± 6.1	29.2 ± 7.7	<0.001
Pain	12.2 ± 1.2	5.0 ± 1.3	4.6 ± 1.5	<0.001
Stiffness	4.7 ± 0.6	1.6 ± 0.6	1.5 ± 0.6	<0.001
Function	42.0 ± 3.2	23.2 ± 4.4	22.7 ± 5.2	<0.001
ΔWOMAC	​	28.7 ± 6.6	29.4 ± 8.0	​
MCID≥12 points	​	40 (100%)	39 (97.5%)	​

Data are presented as mean ± SD, or n (%). P values were calculated using the Friedman test. Abbreviations: WOMAC, Western Ontario and McMaster Universities Osteoarthritis Index.

**FIGURE 4:**
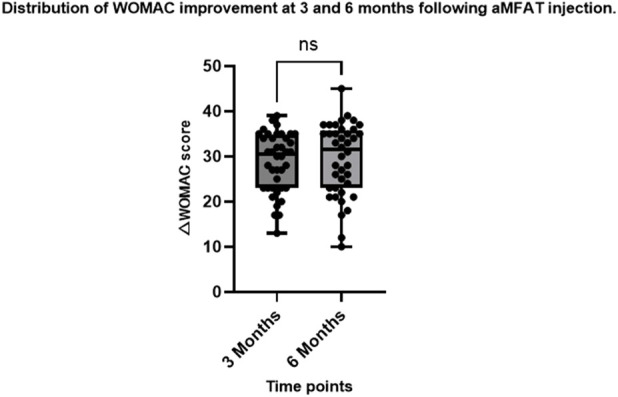
WOMAC improvement was observed at both 3 and 6 months. No significant difference was detected between the two time points (P > 0.05). Boxes represent median; whiskers indicate minimum and maximum values (n = 40).

At 3 months, all patients (100%) achieved the minimal clinically important difference (MCID; WOMAC improvement ≥12 points), while the rate at 6 months was 97.5% (Table 2). Overall, patients demonstrated improvement within 3 months, which was maintained at 6 months (Figure 4).

#### 3.2.2 Secondary outcomes

Compared with baseline, WOMAC pain, stiffness, and function subscale scores were significantly reduced at both 3 and 6 months post-treatment.

### 3.3 Subgroup analysis of cellular parameters and clinical outcomes

Among the 40 patients, 14 underwent cytological analysis. In this subgroup, the median cell viability of the adipose micro-fragmented product was 65.9%, and the median viable nucleated cell load was 9.30 × 10^4^ cells/g.

#### 3.3.1 Cell viability and cell load

At 3 months, the high-viability group showed significantly greater improvement in WOMAC scores compared with the low-viability group (31.9 ± 7.7 vs. 22.3 ± 6.8, P = 0.040). At 6 months, although the high-viability group still demonstrated greater improvement numerically (33.3 ± 9.5 vs. 23.7 ± 8.7), the difference did not reach statistical significance (P = 0.068).

Compared with the low-load group, patients in the high cell load group exhibited more pronounced clinical improvement. The high-load group showed significantly greater improvement at both follow-up time points. At 3 months, the ΔWOMAC score was 33.1 ± 6.0 in the high-load group and 21.0 ± 6.2 in the low-load group (P = 0.008). At 6 months, the difference further increased (36.6 ± 5.6 vs. 20.4 ± 6.0, P = 0.001).

Multivariable regression analyses were performed to evaluate the association between cellular characteristics and clinical outcomes after adjusting for age, sex, body mass index (BMI), Kellgren–Lawrence grade, and baseline WOMAC scores.

After adjustment, higher viable nucleated cell load remained significantly associated with greater improvement in WOMAC scores at both 3 months (B = −10.60, P = 0.012) and 6 months (B = −16.89, P < 0.001). In contrast, cell viability was not significantly associated with clinical outcomes after adjustment at either time point (3 months: B = −4.51, P = 0.400; 6 months: B = −3.17, P = 0.668) (Table 3).

**TABLE 3:** Subgroup analysis according to cellular parameters (n = 14).

Variable	High group	Low group	P value	Adjusted B	P value^*^
Cell viability (%)	75.89 ± 5.54	50.96 ± 11.33	​	​	​
ΔWOMAC (3M)	31.86 ± 7.73	22.29 ± 6.82	0.040	−4.514	0.400
ΔWOMAC (6M)	33.00 ± 9.66	22.86 ± 9.87	0.068	−3.167	0.668
Sustained improvement (%)	6 (85.7%)	4 (57.1%)	​	​	​
Cell load (cells/g)	188,420 ± 81,881	54,655 ± 28,743	​	​	​
ΔWOMAC (3M)	33.14 ± 5.98	21.00 ± 6.16	0.008	−10.603	0.012
ΔWOMAC (6M)	36.57 ± 5.56	19.29 ± 6.75	0.001	−16.891	<0.001
Sustained improvement (%)	7 (100%)	3 (42.9%)	​	​	​

Values are presented as mean ± SD, or n (%). High and low groups were defined based on median values.

* Adjust age, BMI, KL, grade,sex and baseline WOMAC.

**FIGURE 5:**
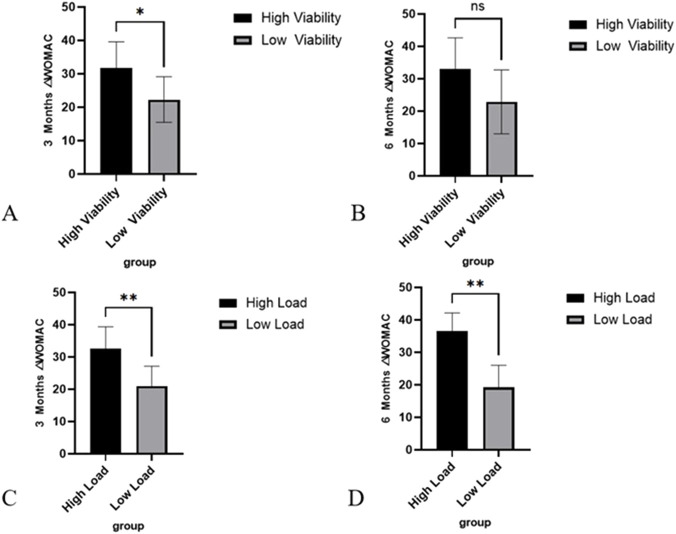
Subgroup analysis of WOMAC total score improvement according to cell viability and viable nucleated cell load. **(A)** Comparison of WOMAC total score improvement between high and low cell viability groups at 3 and 6 months. A significant difference was observed at 3 months (P = 0.040), **(B)**whereas the difference did not reach statistical significance at 6 months (P = 0.068). **(C,D)** Comparison of WOMAC total score improvement between high and low viable nucleated cell load groups at 3 and 6 months. Patients in the high cell load group demonstrated significantly greater improvement at both time points.

These findings suggest that viable nucleated cell load may have a stronger association with clinical outcomes than cell viability alone (Figure 5).

#### 3.3.2 Maintenance of clinical improvement

At the 6-month follow-up, 30 out of 40 patients (75.0%) maintained clinical improvement, whereas 10 patients (25.0%) showed a decline compared with the 3-month follow-up.

**FIGURE 6:**
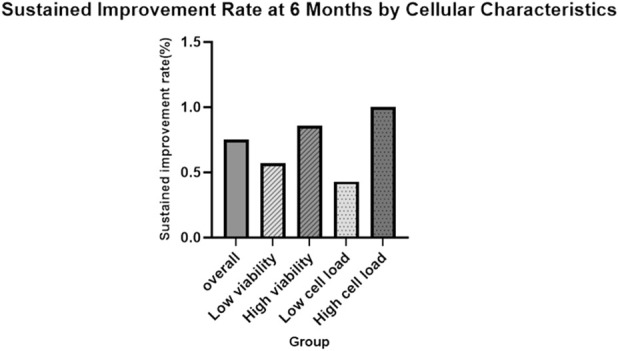
Patients receiving preparations with higher cell viability and higher viable nucleated cell load showed higher rates of sustained clinical improvement.

Subgroup analysis: (1) The proportion of maintained improvement was higher in the high-viability group (85.7%, 6/7) compared with the low-viability group (57.1%, 4/7); (2) All patients in the high-load group (100%, 7/7) maintained improvement, whereas only 42.9% (3/7) of patients in the low-load group did so (Table 3; Figure 6).

#### 3.3.3 Correlation analysis

**FIGURE 7:**
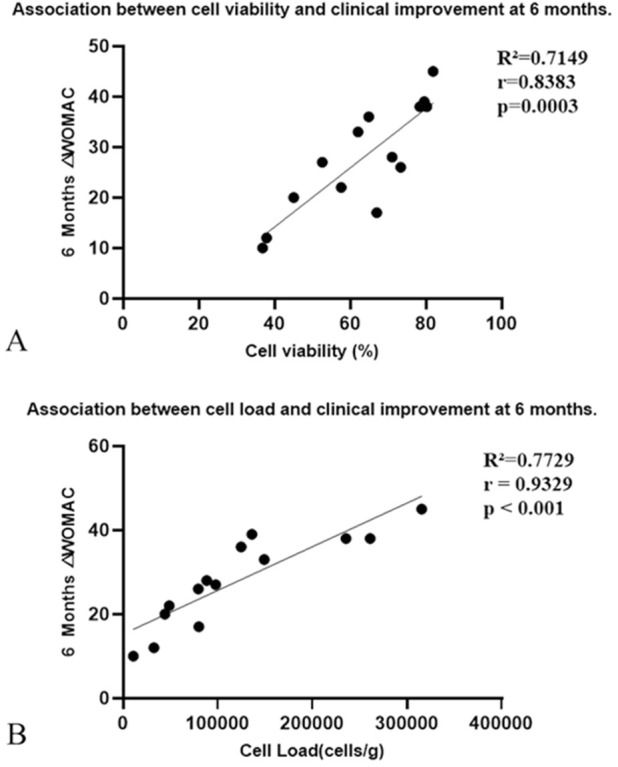
Association between cellular parameters and clinical improvement at 6 months. **(A)** Correlation between cell viability (%) and WOMAC improvement at 6 months. **(B)** Correlation between viable nucleated cell load (cells/g) and WOMAC improvement at 6 months. Spearman’s rank correlation analysis demonstrated significant positive associations between both cellular parameters and clinical improvement.

Spearman correlation analysis showed that cell viability was positively correlated with improvement in WOMAC scores (r = 0.84, P < 0.001), while viable nucleated cell load demonstrated an even stronger correlation (r = 0.93, P < 0.001) (Figure 7).

**FIGURE 8:**
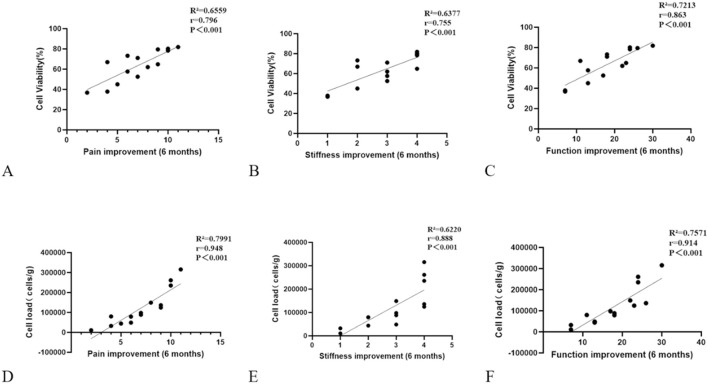
Panels **(A–C)** display corresponding analyses using cell viability (%). Panels **(D–F)** show associations between viable nucleated cell load (cells/g) and improvements in pain, stiffness, and function, respectively. Spearman’s rank correlation analysis revealed consistently stronger associations for cell load across all subscales.

At 6 months, viable nucleated cell load showed strong correlations with improvements in all WOMAC subscales (pain: r = 0.948, stiffness: r = 0.888, function: r = 0.914), which were consistently stronger than those observed for cell viability (Table 4; Figure 8).

**TABLE 4:** Spearman correlation between cellular parameters and clinical improvement.

Variable	3 Months		6 Months	
	r	P value	r	P value
Cell viability	0.883	<0.001	0.868	<0.001
Viable nucleated cell load	0.877	<0.001	0.916	<0.001

#### 3.3.4 Cell subpopulation analysis

In the cell subpopulation analysis, the proportion of fibroblasts showed a significant positive correlation with WOMAC improvement at both 3 and 6 months (P < 0.05), while T cell proportion also demonstrated a positive trend. In contrast, no significant correlation was observed between the proportions of MSCs or macrophages and clinical improvement (P > 0.05) (Table 5).

**TABLE 5:** aMFAT cell subpopulation proportions and correlation with clinical improvement.

Cell population	Mean (SD)	CV	r (3 months)	P value	r (6 months)	P value
MSC	0.29 (0.07)	0.25	−0.225	0.440	−0.253	0.383
T cells	0.13 (0.09)	0.66	0.551	0.041	0.704	0.005
Fibroblasts	0.16 (0.10)	0.63	0.656	0.011	0.752	0.002
Macrophages	0.06 (0.06)	1.06	0.337	0.239	0.321	0.263

CV, coefficient of variation.

### 3.4 Patient satisfaction and adverse events

At the 6-month follow-up, 33 patients (82.5%) reported being satisfied or very satisfied, while 7 patients (17.5%) reported partial satisfaction or dissatisfaction. No serious adverse events were observed (Table 6).

**TABLE 6:** Patient satisfaction after treatment at 6 months (n = 40).

Satisfaction level	n	Percentage (%)
Very satisfied	8	20
Satisfied	25	62.5
Partially satisfied	4	10.0
Dissatisfied	3	7.5

Data are presented as number of patients (n) and percentage (%). Patient satisfaction was assessed at the 6-month follow-up. Satisfaction levels were categorized as very satisfied, satisfied, partially satisfied, and dissatisfied.

A total of 9 patients (22.5%) reported mild and transient adverse events, including knee swelling (5 cases) and temporary pain exacerbation (4 cases), all of which resolved spontaneously within a short period.

## 4 Discussion

This study evaluated the short-term clinical efficacy and safety of a single intra-articular injection of autologous micro-fragmented adipose tissue (aMFAT) in patients with symptomatic knee osteoarthritis, and further analyzed the association between the cellular characteristics of adipose micro-fragmented products and clinical outcomes. The results showed that patients experienced improvement in symptoms as early as 3 months post-treatment, which was maintained at 6 months. In the subgroup undergoing cytological analysis, both cell viability and viable nucleated cell load were positively correlated with clinical improvement, with the latter showing a stronger association. These findings suggest that the clinical benefits of aMFAT therapy may depend not only on the application of the technique itself but also on the intrinsic cellular characteristics of the product.

It is important to note that the magnitude of clinical improvement observed in this study may be influenced by several factors inherent to uncontrolled designs, including placebo effects, regression to the mean, and patient expectation bias. Therefore, the observed improvements should be interpreted with caution.

From a clinical efficacy perspective, the short-term benefits observed in this study are generally consistent with previous reports on adipose-derived cellular therapies for knee osteoarthritis. Richter et al. reported that aMFAT provided superior pain relief compared with saline controls over a 1-year follow-up period (Richter et al., 2025). Comparative studies have shown that aMFAT yields functional improvements comparable to platelet-rich plasma (PRP) (Bąkowski et al. 2020; Varg et al., 2022), although some studies have not demonstrated superiority over placebo (Barfod et al., 2025). Systematic reviews have also indicated that aMFAT or SVF-based therapies have certain clinical value in improving KOA symptoms; however, substantial heterogeneity exists among studies, particularly in terms of product preparation, which remains a major challenge in this field (Hohmann et al., 2025; Hu et al., 2025). Clinical improvement was maintained at 6 months, with no further significant improvement compared with 3 months, suggesting a potential plateau in treatment response. However, given the absence of a control group, the observed improvement may be influenced by placebo effects, regression to the mean, and patient expectation bias, and should therefore be interpreted with caution. As a minimally invasive orthobiologic treatment, aMFAT may represent a feasible option for delaying disease progression in patients with moderate osteoarthritis.

The substantial improvement observed in this study may be partially explained by several factors inherent to uncontrolled designs. First, placebo effects are well documented in osteoarthritis, particularly in studies involving intra-articular interventions, where contextual and procedural factors may enhance perceived symptom relief (Previtali et al., 2025). Second, regression to the mean may contribute to the observed improvement, as patients often seek treatment when symptoms are at their worst, followed by a natural fluctuation toward lower symptom levels over time (Emanuel et al., 2025). Third, patient expectation bias may further influence patient-reported outcomes, especially when individuals are aware that they are receiving a novel biologic therapy, thereby amplifying perceived benefits (Neogi and Colloca, 2023). Together, these factors may partially explain the observed magnitude of improvement.

An important finding of this study is the marked variability in the cellular characteristics of aMFAT products among patients, particularly in terms of cell viability and viable nucleated cell load. This observation is consistent with previous studies reporting heterogeneity in SVF-derived products (Boada-Pladellorens et al., 2022; Richter et al., 2025). Such variability may arise from individual patient factors (e.g., age and metabolic status) as well as differences in tissue harvesting and processing techniques. Previous research has suggested that this biological heterogeneity may contribute to the inconsistent clinical outcomes reported across studies (Richter et al., 2025). Therefore, the ability to rapidly assess the quality of aMFAT products in clinical practice represents an important unmet need.

Furthermore, this study found that viable nucleated cell load was more strongly associated with clinical outcomes than cell viability alone, which has important practical implications. Previous adipose-derived orthobiologics studies have emphasized that these products are not single-cell therapies but rather complex biological constructs, in which overall cell quantity, cellular composition, and preparation methods collectively determine therapeutic efficacy (De Francesco et al., 2021; Bora and Majumdar, 2017). A systematic review by Boada-Pladellorens et al. highlighted substantial variability in cell counts, viability, and concomitant treatments across studies, suggesting that “dose” or “cellular load” may be a key determinant of clinical outcomes (Boada-Pladellorens et al., 2022). Similarly, Russo et al. emphasized the importance of identifying predictors of treatment response in patients receiving aMFAT therapy (Russo et al., 2023). Based on our findings, viable nucleated cell load may better reflect the “biologically active dose” of aMFAT, as it integrates both cell quantity and viability, and may represent a potential exploratory factor related to product quality and clinical outcomes.

In terms of mechanistic insights, this study further explored the relationship between aMFAT cell subpopulations and clinical outcomes. The results showed that the proportion of fibroblasts was positively correlated with WOMAC improvement at both 3 and 6 months, while T cell proportion also showed a positive trend. In contrast, no significant association was observed between MSC proportion and clinical improvement. These findings suggest that aMFAT may function more as a multicellular synergistic system rather than a single MSC-based therapy. Previous studies have proposed that the therapeutic effects ofadipose-derived orthobiologics are partly attributed to its heterogeneous cellular composition and immunomodulatory capacity, with MSCs primarily exerting their effects through paracrine signaling rather than direct tissue replacement (Laver et al., 2024). Within this framework, immune cells may contribute to inflammation regulation, fibroblasts may participate in extracellular matrix remodeling and local microenvironment modulation, and MSCs may act as regulatory mediators. These findings support the concept of aMFAT as a “multicellular regenerative microenvironment carrier” (Kim et al., 2019; Veronesi et al., 2023).

In this context, fibroblasts may play an important role beyond their traditional pro-inflammatory characterization. Recent studies have shown that synovial fibroblasts actively participate in tissue repair and regeneration following joint injury and contribute to maintaining joint homeostasis and supporting cartilage function (Collins et al., 2026; Romano et al., 2023; Xie et al., 2020). Furthermore, fibroblast-related signaling pathways are involved in extracellular matrix remodeling and cartilage homeostasis.

This represents an important and often overlooked aspect of aMFAT-based therapy. While prior studies have predominantly emphasized the role of mesenchymal stem cells (MSCs) in cartilage repair, the potential contribution of other cellular components has received comparatively limited attention. The present findings highlight that non-MSC populations, particularly fibroblasts, may play a critical role in shaping the regenerative microenvironment and influencing clinical outcomes.

This interpretation is also consistent with previous observations that treatment outcomes may be influenced by synovial inflammation and patient-specific factors (Veronesi et al., 2023; Boffa and Filardo, 2025). It is therefore likely that therapeutic efficacy is not determined by the absolute quantity of a single cell type, but rather by the interaction between the cellular components of the product and the joint microenvironment. It should be noted that the cell subpopulation analysis in this study was based on a limited sample size and should be considered exploratory, warranting further validation in future studies.

Optimizing cellular viability and viable nucleated cell load remains an important challenge in aMFAT applications. Although mechanically processed aMFAT offers practical advantages, variability in cellular yield and viability persists. Key factors that may influence product quality include patient-related variability, post-processing quality assessment, and processing techniques.

In terms of safety, no serious adverse events were observed in this study. Only a small number of patients experienced mild and transient knee swelling or pain exacerbation, all of which resolved spontaneously. These findings are consistent with previous systematic reviews of aMFAT, SVF, and adipose-derived MSC therapies, which generally report favorable short-term safety profiles with a low incidence of serious treatment-related adverse events (Kim et al., 2019). Thus, aMFAT appears to be clinically acceptable in terms of safety. Several limitations should be acknowledged. First, this was a single-center retrospective study without a control group, which limits causal inference. Second, the sample size was relatively small, particularly in the cytological subgroup, which was non-randomly selected and may introduce selection bias. Although multivariable analyses were performed, the limited sample size—particularly in the cytological subgroup—may restrict the robustness of adjusted results. Third, the follow-up duration was limited to 6 months, precluding assessment of long-term outcomes. In addition, detailed information regarding patients’ use of medications (e.g., analgesics) and daily activity levels during the follow-up period was not systematically collected or controlled, which may have influenced clinical outcomes. Further prospective studies with larger sample sizes are needed to validate these findings.

## 5 Conclusion

A single intra-articular injection of autologous micro-fragmented adipose tissue (aMFAT) was associated with short-term clinical improvement and a favorable safety profile in patients with knee osteoarthritis. Clinical outcomes showed exploratory associations with cellular characteristics, particularly viable nucleated cell load. These findings require validation in future studies.

## Data Availability

The original contributions presented in the study are included in the article/supplementary material, further inquiries can be directed to the corresponding author.
